# Indirect Carotid Cavernous Fistula with Ocular Manifestations: A Case Report

**DOI:** 10.31729/jnma.8615

**Published:** 2024-06-30

**Authors:** Pooja Shrestha, Angira Shrestha, Tina Shrestha, Richa Makaju Shrestha, Rajani Kesari, Nigi Shrestha, Rahul Gupta

**Affiliations:** 1 Department of Ophthalmology, Kathmandu University School of Medical Sciences, Dhulikhel, Kavre, Nepal

**Keywords:** *carotid-cavernous fistula*, *digital subtraction angiography*, *magnetic resonance imaging*, *embolization*

## Abstract

Carotid-cavernous fistulas are rare entity with incidence of less than 1%, refers to abnormal connections between the carotid artery and cavernous sinus. Indirect types usually occur in elderly female patients and can resolve spontaneously with conservative management like external manual compression of the carotid artery. We report a case of 65-year-old female who presented with complaints of redness, proptosis, chemosis, headache and ophthalmoplegia in her right eye. Digital subtraction angiography revealed Barrow type B indirect carotid-cavernous fistulas. External manual carotid compression was done after which her symptoms improved significantly. Thus, indirect type carotid-cavernous fistulas can occur spontaneously and could be a sight threatening condition especially in elderly females but can resolve with conservative management.

## INTRODUCTION

Carotid-cavernous fistulas (CCFs) are abnormal vascular connections between the internal carotid artery (ICA) or external carotid artery (ECA) and the venous channels of the cavernous sinus.^1^ Direct CCFs most commonly occur after trauma in young males and account for 75% of CCFs and indirect CCFs occur in elderly, hypertensive post-menopausal women and account for 30 % of cases.^2,3^ CCFs usually present with ocular and orbital manifestations thus the Ophthalmologists need to be aware of these symptoms as it may lead to vision loss. We report a case of Right sided indirect CCF of type B with ocular manifestations that could be a diagnostic challenge for all Ophthalmologists.

## CASE REPORT

A 65 year old, known hypertensive female presented with swelling, pain, redness and protrusion of her right eye since five days at Emergency department of our Hospital. Five days prior, she had consulted at a nearby eye hospital and two months back she had seeked neurosurgical advice also when she was prescribed topical and oral analgesics.

**Figure 1:**
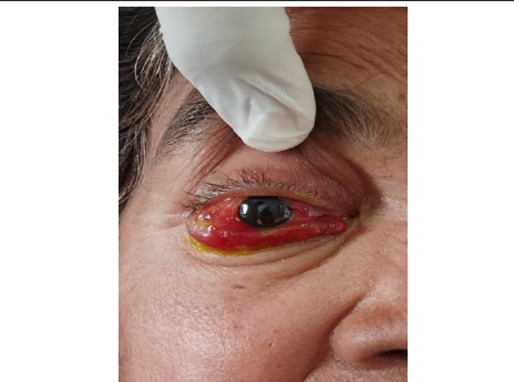
Photograph of the patient showing proptosis and chemosis of right eye at presentation.

At present visit, she had severe headache with nausea, pain and forward protrusion of her right eye. Her best corrected visual acuity (BCVA) was 6/24 in right eye and 6/9 in left eye. In her right eye, she had lid edema, circumciliary congestion, severe conjunctival chemosis in nasal, temporal and inferior quadrants with cork screw type of episcleral vessels (Figure 1).

Anterior chamber was quiet and of adequate depth with no relative afferent pupillary defect (RAPD) but sluggishly reacting pupil. She did not complain of diplopia and orbital bruit was absent on examination. In posterior segment examination, small optic disc with cup disc ratio (CDR) of 0.1, tortous retinal vessels with isolated dot haemorrhage in the supero nasal quadrant were noted. Extraocular movements (EOM) were partially restricted in all the gazes with proptosis of 23 mm in right eye and 21 mm in left eye by Hertel's exophthalmometry. Intraocular pressure (IOP) was 55 mm of Hg in her right eye and 18 mm of Hg in her left eye. Color vision and contrast sensitivity in right eye was impaired. She was admitted and started on intravenous antibiotics ceftriaxone and metronidazole along with anti-glaucoma medications with suspicion of orbital cellulitis.

**Figure 2:**
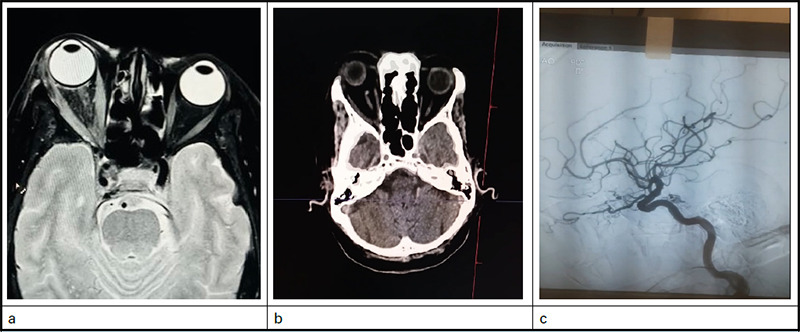
a. MRI showing Proptosis Right eye with extraocular muscle enlargement; b. CT scan showing prominent SOV; c. DSA of patient showing carotid cavernous fistulous connection between left ICA to right cavernous sinus.

Magnetic Resonance Imaging (MRI) of brain and orbit reported: right eye proptosis with altered signal intensity of intraconal fat giving heterogenous appearance with bulky extraconal muscle and thickening of intraconal portion of optic nerve on right side likely inflammatory/infective cause (Figure 2a). So, in the view of inflammatory cause intravenous methylprednisolone was started on the second day of admission. But subsequently, vision deteriorated to finger count close to face in right eye with sluggishly reacting pupil, increasing chemosis, diplopia, IOP of 50 mm of Hg besides multiple anti-glaucoma medications and extra-ocular movements were totally restricted. In posterior segment examination, optic disc had CDR of 0.1, with increased tortuosity of retinal vessels and blot haemorrhage in the supero nasal quadrant. MRI was repeated but revealed similar findings. So, with the suspicion of aterio-venous malformations, CT venogram and arteriogram were performed on the eighth day (Figure 2b). Right superior ophthalmic vein was notably dilated with normal cavernous sinus without evidence of filling defect so on eleventh day, digital subtraction angiography (DSA) was performed after neurosurgical consultation which suggested indirect CCF of type B Barrow classification with fistulous connection from left ICA to right cavernous sinus (Figure 2c). They suggested for frequent right sided external carotid compression with left hand and planned for coiling if condition of the patient deteriorated. But subsequently vision improved to 6/36 in the affected eye with decrease of conjunctival chemosis and normalization of IOP so oral steroids and antibiotics were tapered.

**Figure 3:**
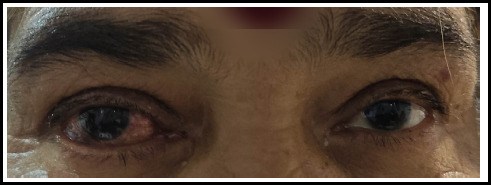
Resolved conjunctival chemosis final follow up.

Finally, on 16th day, patient was discharged with vision of 6/12 in her right eye with resolved conjunctival chemosis, improved range of extraocular movements and controlled IOP with advice of continual of right sided carotid massage and anti-glaucoma medications. (Figure 3) Patient has been on regular follow up and planned for coiling or embolization of CCF if recurrence of the symptoms.

## DISCUSSION

CCFs have been classified based on hemodynamic properties, etiology and anatomy of the fistulas. Anatomically direct CCFs arise directly from carotid artery while indirect ones arise from carotid artery branch vessels.^4^ Barrow et al have defined four types (type A-D) of CCFs. Type A CCFs are high flow lesions connecting internal carotid artery directly with cavernous sinus whereas type B, C and D are indirect low flow lesions that arise from the meningeal branches of internal or external carotid artery.^5^ Type A CCFs are the commonest and account for 75-80% of overall CCF whereas other types are rare.^6^ In a study conducted among 132 patients with CCF, 75.8% were of type A, 21.6% were type D, 3% were of type C and none were of type B.^6^ Our patient was elderly hypertensive female which is a risk factor for indirect CCF had type B CCF involving meningeal branch of ICA with proptosis, chemosis and ophthalmoplegia. Other studies also have reported 72-98% of proptosis, chemosis in 55100%, orbital bruit in 71-80%, ophthalmoplegia in 2363% and diplopia in 88%.^6-10^

Our patient was initially treated with empiric broad spectrum antibiotics for suspected orbital cellulitis. However, due to lack of clinical improvement with normal thyroid function tests, normal inflammatory markers and enlarged SOV in MRI orbit demanded alternative diagnosis. Finally, DSA procedure was conducted which revealed type B indirect CCF in our patient with fistulous connection from left ICA to right cavernous sinus which improved with external carotid artery manual compression.

Digital subtraction angiography (DSA) is known to be gold standard modality for the diagnosis of CCF. The goal of CCF treatment is to completely occlude the fistula, preserving the normal blood flow through ICA. Similar to our case, conservative management like external manual compression of ipsilateral carotid artery several times a day for four to six weeks has been tried successfully in the treatment of indirect CCFs in other cases also.^4^ Success rate of this compression has been reported to be 35% with resolution occurring between two weeks and seven months after initiation.^11^ However, 20-60% of cases of indirect CCFs have been reported to have spontaneous fistula closure.^4^ Previously, ligation of CCA was surgical treatment of choice for CCF but in present era, trans arterial or trans venous embolization with metallic coils or liquid embolic agents is the treatment of choice.^8,10^

Indirect CCFs are rare but can occur spontaneously and could be a sight threatening condition. Ophthalmologists should be familiar with the symptoms. Treatment should be initiated early to preserve vision and long term follow up is demanded because of possibility of recurrence of the disease.
